# Computer-aided diagnosis for screening of lower extremity lymphedema in pelvic computed tomography images using deep learning

**DOI:** 10.1038/s41598-023-43503-1

**Published:** 2023-09-27

**Authors:** Yukihiro Nomura, Masato Hoshiyama, Shinsuke Akita, Hiroki Naganishi, Satoki Zenbutsu, Ayumu Matsuoka, Takashi Ohnishi, Hideaki Haneishi, Nobuyuki Mitsukawa

**Affiliations:** 1 Center for Frontier Medical Engineering, Chiba University, 1-33 Yayoi-Cho, Inage-ku, Chiba, 263-8522 Japan; 2 Department of Medical Engineering, Faculty of Engineering, Chiba University, 1-33 Yayoi-Cho, Inage-ku, Chiba, 263-8522 Japan; 3 Department of Plastic, Reconstructive and Aesthetic Surgery, Graduate School of Medicine, Chiba University, 1-8-1 Inohana, Chuo-ku, Chiba, 260-8670 Japan; 4 Department of Plastic Surgery, Saiseikai Yokohamashi Nanbu Hospital, 3-2-10 Konandai, Konan-ku, Yokohama City, Kanagawa 234-0054 Japan; 5 Department of Gynecology and Maternal-Fetal Medicine, Chiba University Hospital, 1-8-1 Inohana, Chuo-ku, Chiba, 260-8670 Japan; 6 Department of Pathology and Laboratory Medicine, Memorial Sloan Kettering Cancer Center, 1133 York Avenue, New York, NY 10065 USA

**Keywords:** Biomedical engineering, Computed tomography, Oedema

## Abstract

Lower extremity lymphedema (LEL) is a common complication after gynecological cancer treatment, which significantly reduces the quality of life. While early diagnosis and intervention can prevent severe complications, there is currently no consensus on the optimal screening strategy for postoperative LEL. In this study, we developed a computer-aided diagnosis (CAD) software for LEL screening in pelvic computed tomography (CT) images using deep learning. A total of 431 pelvic CT scans from 154 gynecological cancer patients were used for this study. We employed ResNet-18, ResNet-34, and ResNet-50 models as the convolutional neural network (CNN) architecture. The input image for the CNN model used a single CT image at the greater trochanter level. Fat-enhanced images were created and used as input to improve classification performance. Receiver operating characteristic analysis was used to evaluate our method. The ResNet-34 model with fat-enhanced images achieved the highest area under the curve of 0.967 and an accuracy of 92.9%. Our CAD software enables LEL diagnosis from a single CT image, demonstrating the feasibility of LEL screening only on CT images after gynecologic cancer treatment. To increase the usefulness of our CAD software, we plan to validate it using external datasets.

## Introduction

Lymphedema is a chronic and progressive condition characterized by the accumulation of protein-rich fluid in the interstitial spaces because of lymphatic system mechanical insufficiency, which can result in soft tissue swelling, chronic inflammation, reactive tissue fibrosis, and abnormal adipose tissue deposition^1–3^. While it can affect any body part, it commonly occurs in the extremities, such as the arms and legs. Advanced lymphedema causes irreversible changes in the skin and soft tissues, and conservative treatment, such as compression therapy, must be continued throughout life^1^.

Lymphedema is classified into primary and secondary forms^2, 3^. Primary lymphedema is caused by innate abnormalities in lymphatic system development, leading to underdeveloped or malfunctioning lymphatics. It can develop in infancy, puberty, or even later in life. Secondary lymphedema is caused by damage or obstruction of previously normal lymphatics by external factors such as surgery, radiation therapy, infection, or traumatic damage. Approximately 99% of all lymphedema patients suffer from secondary lymphedema^2^. Filarial infection remains the leading cause of secondary lymphedema in developing countries^3^. In contrast, cancer-related treatment is the most common cause of secondary lymphedema in developed countries.

Lower extremity lymphedema (LEL) is a common complication after gynecological cancer treatment^4, 5^. The reported prevalence of postoperative LEL varies with the diagnostic test used. A systematic review by Bona et al. reported the incidence of LEL after cervical cancer varied between 0 and 69%^6^. Secondary LEL after gynecologic cancer treatment is a significant iatrogenic quality of life complication, and the risk of developing cellulitis, lymphorrhea, and cosmetic problems of leg deformities is lifelong. This condition affects the patient’s activities of daily living and quality of life^7, 8^. Early diagnosis and timely intervention can prevent further LEL development and even more severe complications^9^. However, gynecologists are not experts in lymphedema, so establishing a screening strategy that provides a bridge to specialists can help. Hence, it is required to establish a screening strategy for LEL. However, there is currently no consensus on the optimal screening strategy for postoperative LEL^5, 10^.

Currently, there are several methods to diagnose LEL, such as limb circumference measurement^11^, bioimpedance spectroscopy (BIS)^12^, gynecologic cancer lymphedema questionnaire (GCLQ)^13^, lymphoscintigraphy^14^, and indocyanine green (ICG) lymphography^15^ to assess and diagnose LEL. Limb circumference measurement is the traditional method for calculating limb volume by measuring circumferences at predetermined, short intervals along the limb. However, it is time-consuming, and accurate measurement is difficult to perform except by experts, and the number of experts is small. BIS measures the resistance met by the low-frequency electrical current. BIS measurements detect extracellular fluid volume differences between limbs by measuring the impedance of electrical flow that passes through a body section. Although it has been increasingly used to screen for breast cancer-related lymphedema, further studies are needed to determine its utility in LEL related to gynecologic cancers^10^. The GCLQ can be easily incorporated into clinical settings to monitor potential LEL symptoms. However, the GCLQ combined with circumferential measurements may be required to improve the ability to diagnose LEL^13^. Lymphoscintigraphy and ICG lymphography are useful tools for the diagnosis of lymphatic function, but they are accompanied by pain owing to subcutaneous injection.

We focused on pelvic computed tomography (CT) scans performed after gynecological cancer treatment. Our previous study proposed a novel lymphedema screening method based on the subcutaneous fat thickness in pelvic CT images before and after cancer surgery^16^. In this method, the thickness of the subcutaneous fat layer on the lateral edge of the rectus femoris muscle at the level of the lesser trochanter of the femur is measured. The perioperative temporal subcutaneous fat thickness index, which is calculated by dividing the thickness after surgery by the thickness after surgery, is used as an assessment criterion. This assessment index is useful for screening early-stage lymphedema. However, this method requires CT scans before and after the surgery. Moreover, it is difficult to exclude the effects of weight changes and other factors. Therefore, it is desirable to realize LEL screening only in CT images after cancer treatment.

Computer-aided detection/diagnosis (CAD) software, which includes the classification of lesions and the detection of lesions on the image, has been developed by many research groups, and CAD software using deep learning has dramatically increased in recent years^17–19^. We expect to achieve LEL screening by applying deep learning to pelvic CT images after cancer treatment. This study aimed to realize a CAD software for LEL screening in pelvic CT images using deep learning.

## Results

We used 431 pelvic CT images from 154 patients for follow-up after gynecological cancer treatment at Chiba University Hospital, Chiba, Japan. The detail of the dataset is described in Methods. All patients underwent ICG lymphography and were staged by one experienced plastic surgeon (S.A.) according to the six-stage classification proposed by Yamamoto et al.^15^ for each leg. In this study, a patient with ICG dermal backflow pattern (DBF) stage II or higher in either leg was considered positive, and a patient with ICG DBF stage 0 or I in both legs was considered negative. Positive and negative examinations were 205 and 226. These data were randomly divided into three subsets: training, validation, and test sets (Table 1). The training set was used to train the model. The validation set was used to calculate the evaluation criterion of hyperparameter tuning. The test set was used to evaluate the best model throughout the hyperparameter tuning. All CT images from the same patient were assigned to the same subset. The demographic characteristics among ICG DBF stages are shown in Supplemental Table S1.

**Table 1:** Demographic characteristics in the training, validation, and test sets.

Label	Training set	Validation set	Test set	Total
Negative				
No. of examinations	161	30	35	226
Age (year)^a^	57 ± 12	58 ± 6	53 ± 10	57 ± 11
Sex				
Male	0 (0.0)	0 (0.0)	0 (0.0)	0 (0.0)
Female	161 (100.0)	30 (100.0)	35 (100.0)	154 (100.0)
Cancer type				
Cervix	17 (10.6)	13 (43.3)	7 (20.0)	37 (16.4)
Uterine corpus	64 (39.7)	15 (50.0)	8 (22.9)	87 (38.5)
Ovary and tube	77 (47.8)	2 (6.7)	20 (57.1)	99 (43.8)
Vulva and others	3 (1.9)	0 (0.0)	0 (0.0)	3 (1.3)
Positive				
No. of examinations	140	30	35	205
Age (year)^a^	59 ± 12	55 ± 11	58 ± 13	58 ± 12
Sex				
Male	0 (0.0)	0 (0.0)	0 (0.0)	0 (0.0)
Female	140 (100.0)	30 (100.0)	35 (100.0)	154 (100.0)
Cancer type				
Cervix	62 (44.3)	17 (56.7)	13 (37.1)	92 (44.9)
Uterine corpus	31 (22.1)	6 (20.0)	7 (20.0)	44 (21.4)
Ovary and tube	46 (32.9)	7 (23.3)	12 (34.3)	65 (31.76)
Vulva and others	1 (0.7)	0 (0.0)	3 (8.6)	4 (2.0)
Affected side of lymphedema				
Right	41 (29.3)	9 (30.0)	12 (34.3)	62 (30.2)
Left	26 (18.6)	9 (30.0)	9 (25.7)	44 (21.5)
Both	73 (52.1)	12 (40.0)	14 (40.0)	99 (48.3)

Except where indicated, data are numbers of patients, with percentages in parentheses.

^a^Data are mean ± standard deviation.

**Figure 1:**
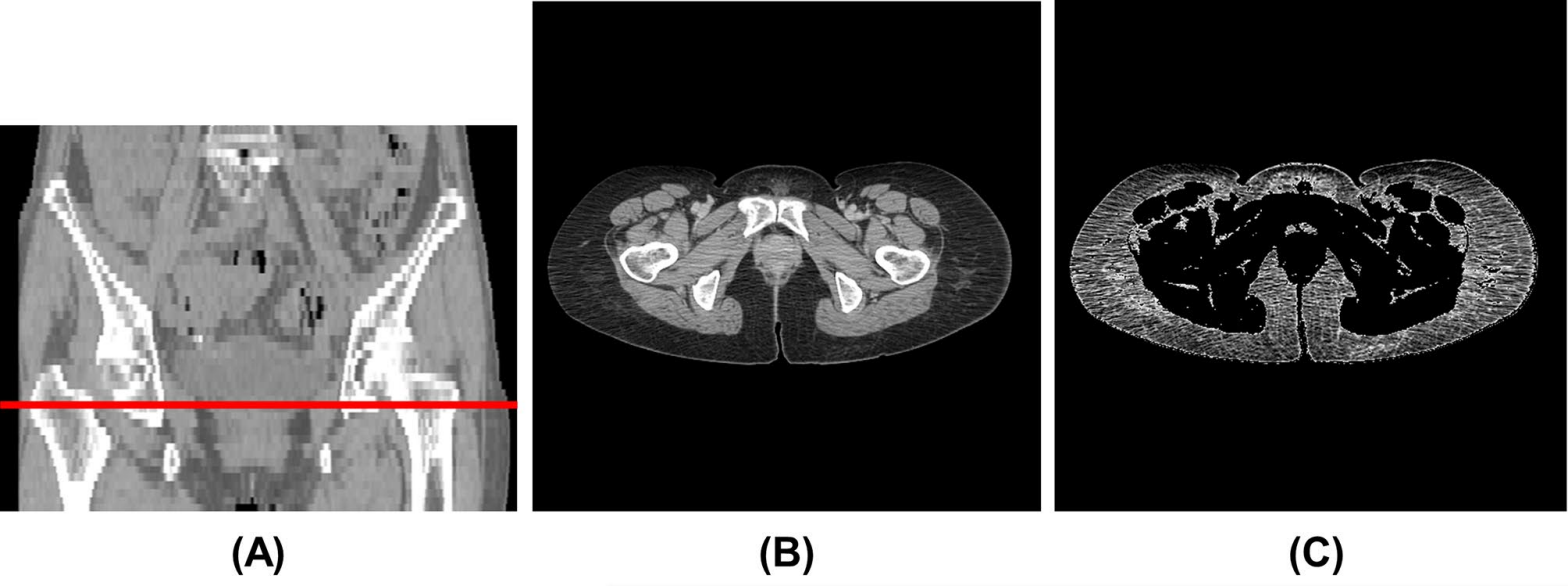
Example of CT image at the greater trochanter level. (**A**) Coronal section (red line: greater trochanter level), (**B**) original image (window level, 50 HU; window width, 400 HU), (**C**) fat-enhanced image.

We used the CT image at the greater trochanter level (Fig. 1A,B) as the input image for the convolutional neural network (CNN) model since it was included in the imaging range of the pelvic CT scan and was close to the inguinal lymph nodes. One radiological technologist (Y.N.) identified the slices at the greater trochanter level. The body trunk region was extracted through thresholding and morphological processing to eliminate the CT table and other non-body objects from the CT images. After that, a fat-enhanced image (Fig. 1C) corresponding to the fat region was created as follows:

1 $${I}_{fat}\left(\mathbf{x}\right)=\left\{\begin{array}{ll}0.01\times \left\{I\left(\mathbf{x}\right)-125\right\}, & -125\le I\left(\mathbf{x}\right)\le -25\\ 0, & \rm{otherwise}\end{array}\right.$$

where $I\left(\mathbf{x}\right)$ is the CT value (HU) and $\mathbf{x}$ is the two-dimensional coordinates of the pixel. The threshold values in Eq. (1) were determined experimentally based on the fact that the CT value range for adipose tissue was − 140 to – 30 HU^20^. As a comparison, we also evaluated the case where the original image was used as input of the CNN model. The original images were normalized to soft-tissue window (window level, 50 HU; window width, 400 HU).

The ResNet-18, ResNet-34, and ResNet-50 models^21^ were employed as the CNN model. The input size of the models was set to 512 × 512 pixels, and the models were trained from scratch. We used the momentum stochastic gradient descent with a weight decay of 0.0001 and momentum of 0.9 to optimize the network weights. The training procedure of the CNN model is described in Methods.

**Figure 2:**
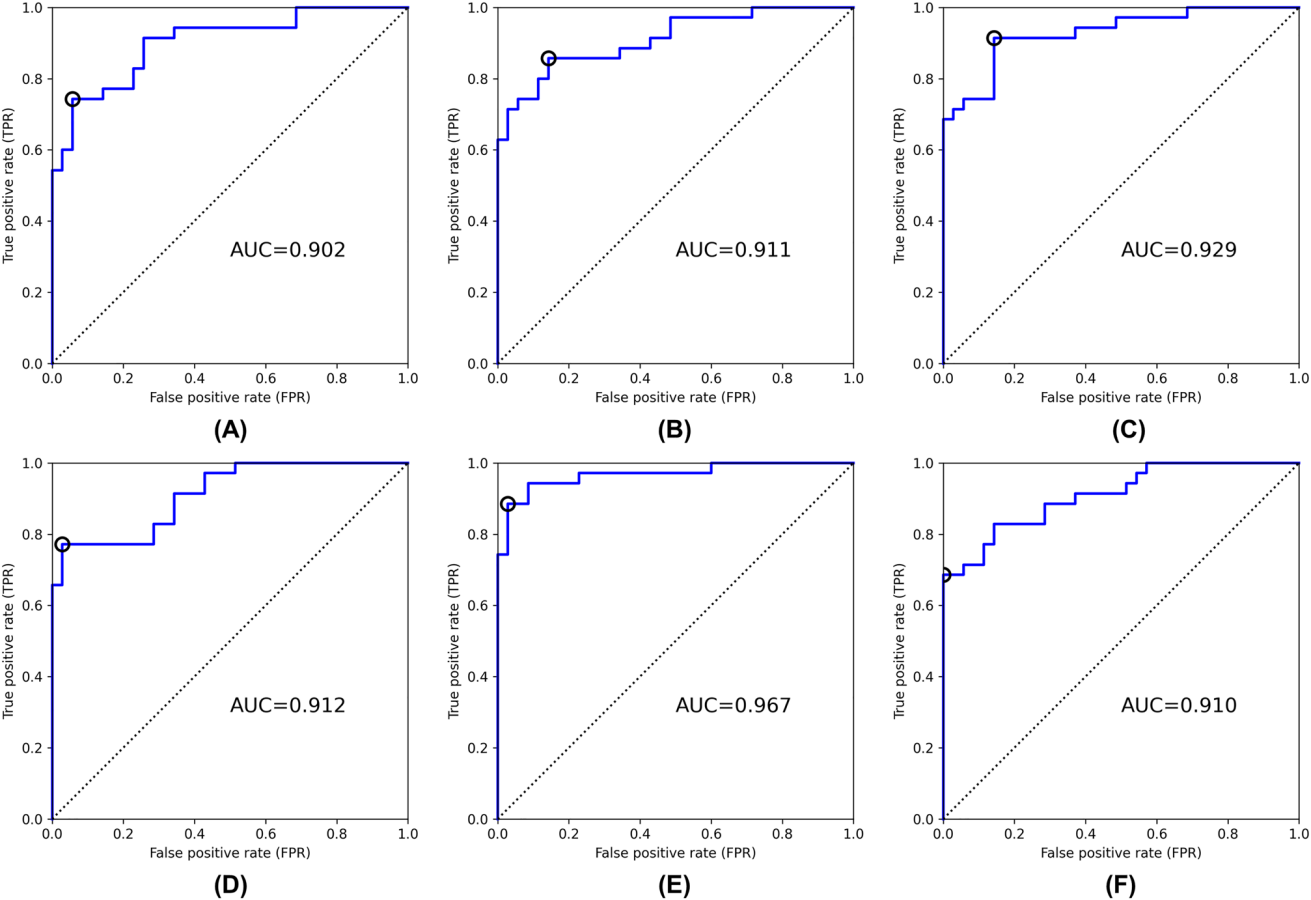
ROC curves for test set for each input image and model [(**A**) original image, ResNet-18, (**B**) original image, ResNet-34, (**C**) original image, ResNet-50, (**D**) fat-enhanced image, RedNet-18, (**E**) fat enhanced image, ResNet-34, (**F**) fat-enhanced image, ResNet-50]. The circle indicates the cutoff point chosen using Youden’s index. AUC: area under the curve.

Receiver operating characteristic (ROC) analysis was used to evaluate our method^22, 23^, and the area under the curve (AUC) was calculated. Figure 2 shows the ROC curves for each input image and model. Table 2 shows classification performance at the optimal cutoff point for each input data. Youden’s index^24^ was used to select the optimal cutoff point of the ROC curve. The ResNet-34 with fat-enhanced images yielded the highest AUC of 0.967 and an accuracy of 0.929, as shown in Fig. 2 and Table 2. By comparing the input images, the fat-enhanced image demonstrated superior classification performance for each criterion over the original image in both ResNet-18 and ResNet-34 models.

**Table 2:** Classification performance at the optimal cutoff point for each input image and each model.

Input image	Model	Accuracy	Sensitivity	Specificity	Positive predictive value	Negative predictive value
Original	ResNet-18	0.843	0.743	0.943	0.929	0.786
	ResNet-34	0.857	0.857	0.857	0.857	0.857
	ResNet-50	0.886	0.914	0.857	0.865	0.909
Fat-enhanced	ResNet-18	0.871	0.771	0.971	0.964	0.810
	ResNet-34	0.929	0.886	0.971	0.969	0.895
	ResNet-50	0.843	0.686	1.000	1.000	0.761

**Figure 3:**
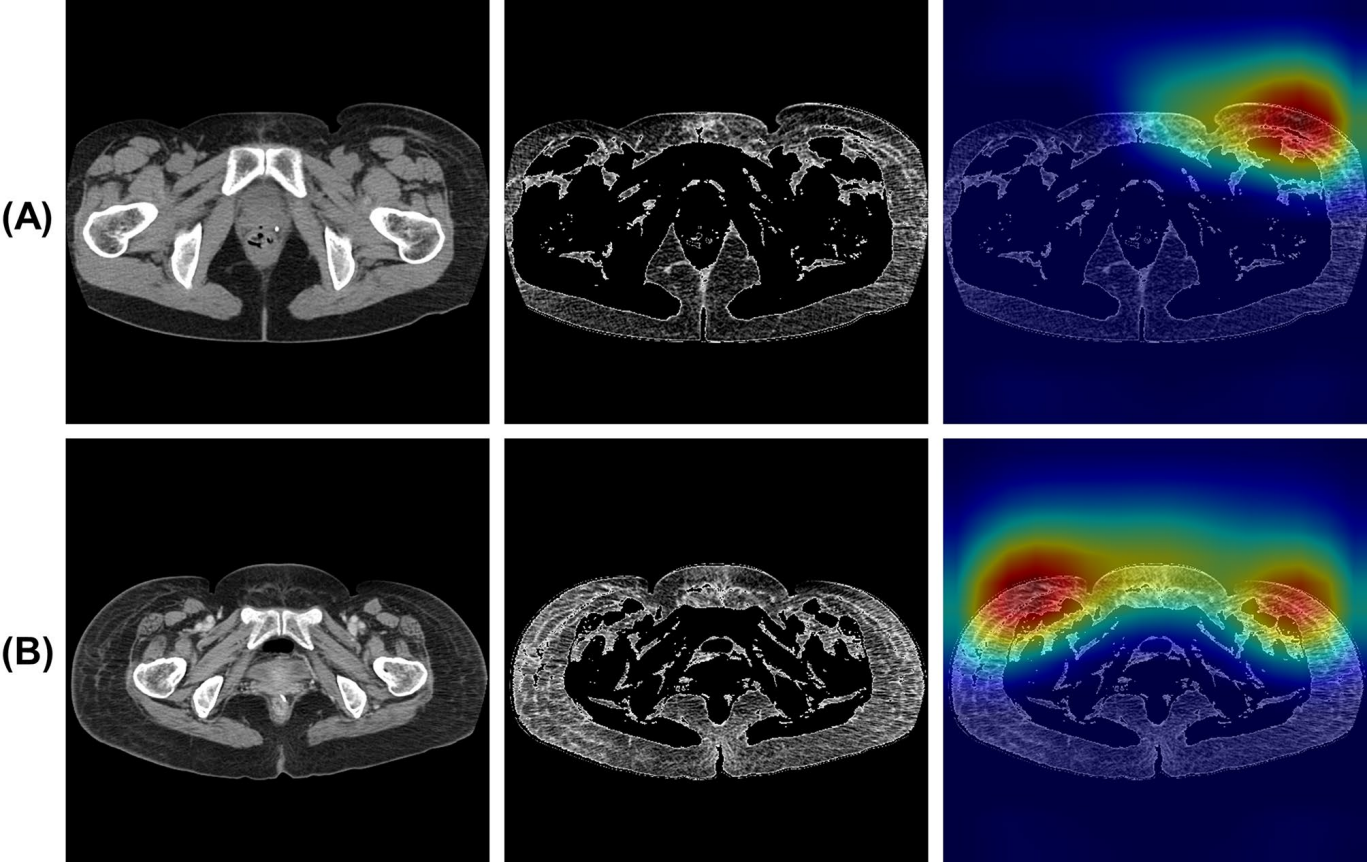
Examples of correctly classified as positive [(**A**) affected side: left, (**B**) affected side: both]. From left to right, original image, fat-enhanced image, and Grad-CAM heatmap overlaid on the fat-enhanced image.

**Figure 4:**
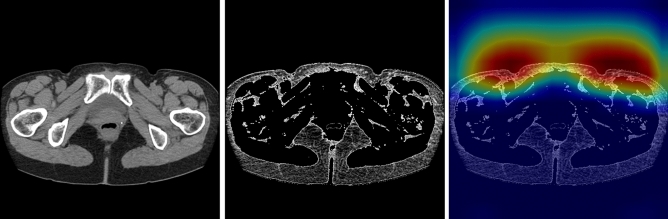
Example of incorrectly classified as positive (normal case). From left to right, original image, fat-enhanced image, and Grad-CAM heatmap overlaid on the fat-enhanced image.

**Figure 5:**
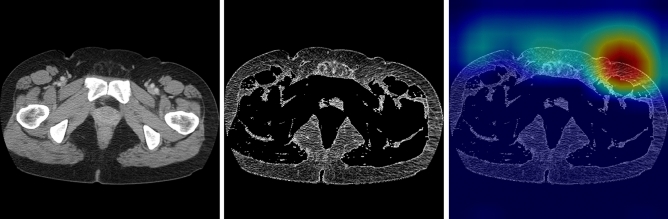
Example of incorrectly classified as negative (affected side: right). From left to right, original image, fat-enhanced image, and Grad-CAM heatmap overlaid on the fat-enhanced image.

Figures 3, 4 and 5 show examples of gradient-weighted class activation mapping (Grad-CAM)^25^ images that visualize the rationale for classification using the ResNet-34 model with the fat-enhanced image. Figure 3 shows examples of the case correctly classified as positive, with the Grad-CAM heatmaps indicating that the anterolateral subcutaneous adipose region on the affected side contributes to the classification. Figure 4 shows an example of a negative case misclassified as positive, and the corresponding Grad-CAM heatmap shows the contribution of the anterolateral subcutaneous adipose regions on both sides to the classification. Figure 5 shows a positive case with lymphedema in the right leg that was misclassified as negative, and the Grad-CAM heatmap from this figure reveals the contribution of the contralateral anterolateral subcutaneous adipose region on the left side to the classification.

## Discussion

We have successfully developed a CAD software for LEL screening in pelvic CT images using deep learning. Our method enables LEL diagnosis from a single CT image at the greater trochanter level after gynecologic cancer treatment. The results indicate that the ResNet-34 model with a fat-enhanced image achieved an AUC of 0.967 and an accuracy of 92.9%. In the ResNet-18 and ResNet-34 models, the fat-enhanced image yielded superior classification performance compared to the original image because of its emphasis on alterations in the subcutaneous fat layer and masking of other regions, such as muscle and bone. In contrast, the performance of the ResNet-50 model trained with fat-enhanced images is inferior to that of the ResNet-50 model trained with original images. Because the fat-enhanced image is masked in other regions, excessive complexity may degrade performance. This result is similar to previous studies in which a shallower model produced superior performance^26, 27^. Hence, this study demonstrates the feasibility of LEL screening only on CT images after gynecologic cancer treatment.

Our classification model detects LEL based on the presence of organic changes in anterior subcutaneous adipose tissue, such as fibrosis, as the Grad-CAM images in Figs. 3, 4 and 5. This is in alignment with our previous method^16^, which detected LEL through the assessment of subcutaneous fat thickness in pelvic CT images. However, the negative case demonstrating organic changes in anterior subcutaneous adipose tissue due to other factors led to misclassification. Additionally, the positive case without organic changes in anterior subcutaneous adipose tissue resulted in a false negative. Inputting a possible way to further improve performance would be to input cropped images of the anterolateral subcutaneous adipose regions of each side into the model.

In our previous study, we demonstrated that ICG DBF stage I refers to temporary changes in examination results that can potentially improve over time through natural progression^28^. In contrast, ICG DBF stage II represents chronic findings that did not improve through natural progression. This indicates that ICG DBF stages 0 and I represent imaging findings consistent with stage 0 of the International Society of Lymphology (ISL) Clinical Staging System, while ICG DBF stage II or higher corresponds to the stage I or higher of the ISL Clinical Staging System, which shows pathological changes that need treatments. On the other hand, although the ISL Clinical Staging System is widely used in the world, its definition is subjective and lacks precision. Therefore, in this study, we defined positivity as ICG DBF stage II or higher, which objectively indicates the state of chronic lymphedema requiring treatment.

The National Comprehensive Cancer Network guidelines do not recommend follow-up CT scans for asymptomatic patients after gynecologic cancer treatment^29–31^. By contrast, they are recommended when symptoms are present or recurrence/metastasis is suspected. In cervical cancer, positron emission tomography (PET)/CT or CT scan is recommended within 3–6 months following initial treatment of advanced cases and for follow-up in poor prognosis histology^31^. In ovarian cancer, radiographic imaging, including CT, whole-body magnetic resonance imaging, or PET/CT, should be conducted periodically if the CA-125 level is normal at the onset of treatment^32^. Hence the application of our CAD software to patients after gynecological cancer treatment is limited. On the other hand, Wong et al. reported a 6.8% prevalence of undiagnosed LEL after gynecologic cancer treatment^5^. The number of undiagnosed LEL is expected to reduce using our CAD software if a follow-up CT scan is conducted after gynecologic cancer treatment.

Recently, several research groups have reported on lymphedema detection based on machine learning^33, 34^. Fu et al. proposed an artificial neural network model for early detection of lymphedema in breast cancer patients based on real-time self-reported symptoms. The model achieved accuracy, sensitivity, and specificity of 93.75%, 95.65%, and 91.03%, respectively^33^. Wei et al. proposed a symptom-warning model for early detection of breast cancer-related lymphedema. The model was developed by logistic regression with 24 lymphedema-associated symptoms and achieved accuracy sensitivity, and specificity of 82.5%, 77.1%, and 88.3%, respectively^34^. A similar method using machine learning based on symptoms would be developed for LEL detection after gynecologic cancer treatment. In addition, integrating this method with our CAD software may provide a more optimal screening method for postoperative LEL. Several studies have been reported on performance improvement in prediction or CAD by integrating machine learning models^35–37^.

The limitations of this study are as follows. First, all CT images were sourced from a single institution, with the majority of patients being racially homogeneous. Hence, the results may not be universally applicable across different racial groups. The collection of additional data for training the deep learning model is essential to accommodate a wider range of populations. In addition, we plan to validate our CAD software using external datasets. Second, all the patients were females who had undergone after gynecologic cancer treatment. However, cancer-related LEL has also been observed in other types of cancer, including melanoma, colorectal cancer, and prostate and penis cancers. Therefore, it is necessary to validate our CAD software’s applicability to CT images after treatment of these cancers, as well as in male patients. This step will improve the utility of our CAD software. Third, the input CT images at the greater trochanter level were manually selected. The implementation of an automated selection method from CT images is necessary for our CAD software to be practically useful in a clinical setting. Fourth, we used the ResNet model as the deep learning model. The performance of our CAD software can be improved by using a novel deep learning model, such as vision transformers^38, 39^.

In conclusion, we have realized the CAD software for LEL screening in pelvic CT images using deep learning. Our CAD software enables LEL diagnosis from a single CT image, demonstrating the feasibility of LEL screening only on CT images after gynecologic cancer treatment.

## Methods

### Datasets

This study was approved by the research ethics committee of the Graduate School of Medicine, Chiba University, Chiba, Japan (Approval Number M10235, Mar. 10, 2022, and Approval Number M10291, July 27, 2022). All the following procedures were in accordance with the ethical standards of the institutional research committee and with the 1975 Helsinki declaration and its later amendments or comparable ethical standards. Due to the retrospective study design, informed consent was waived by the research ethics committee of the Graduate School of Medicine, Chiba University. We selected 433 pelvic CT images from 155 patients for follow-up after gynecological cancer treatment at Chiba University Hospital, Chiba, Japan, from January 2008 to February 2018 according to the following criteria: (1) The slice thickness of the CT image was 5.0 mm, (2) Lymphatic function was assessed by ICG lymphography by injecting 0.2 ml of subcutaneous ICG into the first web space of both lower limbs. Two CT images from a single patient undergoing artificial femoral head replacement were excluded. In total, 433 pelvic CT images from 154 patients were used in this study. Nine CT scanners (Activion 16, Aquilion, Aquilion 64, Aquilion ONE, Aquilion PRIME, and Alexion, Canon Medical Systems Corporation, Otawara, Japan; Brilliance 40, Philips Healthcare, Best, the Netherlands; SOMATOM Definition AS+, Siemens Healthcare, Erlangen, Germany; Brivo CT385, GE Healthcare, Waukesha, WI, USA) were employed for these pelvic CT scans. The scan parameters for the pelvic CT images were as follows: field of view, 280–500 mm; matrix size, 512 × 512 pixels; pixel spacing, 0.546–0.976 mm; slice thickness: 5.0 mm. Of the 431 examinations, 234 were contrast-enhanced.

### Training of CNN

We trained each CNN model from scratch on original and *K* augmented images for each image. The augmented images were generated through a random rotation within ± 30° as well as a random horizontal flip with 50% probability and were changed for each epoch. We carried out ten trials of hyperparameter tuning with the random search^40^ for each input image and model. The tuned hyperparameters of the CNN were the batch size (10–40, step: 2), learning rate (10^–3^–10^–6^), and the ratio of dropout (0–0.5), the number of data augmentations for each image (*K* = 1–3). We utilized the AUC of the ROC curve as an evaluation criterion for hyperparameter tuning. The numbers of maximum epochs and the patience of early stopping^41^ were set to 600 and 20, respectively.

PyTorch^42^ version 1.12.1 was used to implement the CNN model. We trained the model on an RTX A6000 (NVIDIA Corporation, Santa Clara, CA) graphics processing unit with 48 GB memory. The selected sets of hyperparameters for the CNN model, corresponding to each input data set, are shown in Table 3.

**Table 3:** Selected sets of hyperparameters of the CNN model for each input image and each model.

Input image	Model	Batch size	Learning rate	Number of data augmentations for each image
Original	ResNet-18	10	0.0009568	1
	ResNet-34	26	0.0004327	2
	ResNet-50	16	0.0005644	1
Fat-enhanced	ResNet-18	24	0.0001120	3
	ResNet-34	12	0.0001555	2
	ResNet-50	36	0.0003706	3

### Supplementary Information

Supplementary Table S1.

## Data Availability

The datasets generated and/or analyzed during the current study are not publicly available due patient privacy regulations but are available from the corresponding author on reasonable request.
