# Supplementary material for: Computer-aided diagnosis for screening of lower extremity lymphedema in pelvic computed tomography images using deep learning
Source: Sci Rep. 2023 Sep 27;13:16214. doi: 10.1038/s41598-023-43503-1 (PMC10533488; doi:10.1038/s41598-023-43503-1)
Supplement: Supplementary file 1 — Supplementary Table S1. [file 41598_2023_43503_MOESM1_ESM.pdf]

Table S1 Demographic characteristics among Indocyanine green dermal backflow stages.

Label	Affected side of lymphedema	ICG DBF stage (right, left)	Training set	Validation set	Test set	Total
Negative		(0, 0)	112	26	28	166
		(0, I)	4	4	4	12
		(I, 0)	20	0	3	23
		(I, I)	25	0	0	25
Positive	Right	(II, 0)	5	6	5	16
		(II, I)	13	0	0	13
		(III, 0)	9	0	4	13
		(III, I)	8	1	3	12
		(IV, 0)	10	1	0	11
		(IV, I)	1	0	0	1
		(V, 0)	0	0	0	0
		(V, I)	0	0	0	0
	Left	(0, II)	7	4	4	15
		(0, III)	10	0	3	13
		(0, IV)	0	0	1	1
		(0, V)	0	0	1	1
		(I, II)	0	5	0	5
		(I, III)	7	0	0	7
		(I, IV)	1	0	0	1
		(I, V)	1	0	0	1
	Both	(II, II)	15	4	6	25
		(II, III)	22	0	0	22
		(II, IV)	1	0	1	2
		(II, V)	0	0	0	0
		(III, II)	3	2	3	8
		(III, III)	18	1	2	21
		(III, IV)	0	0	1	1
		(III, V)	0	0	0	0
		(IV, II)	3	0	0	3
		(IV, III)	1	0	1	2
		(IV, IV)	0	4	0	4
		(IV, V)	0	1	0	1
		(V, II)	0	0	0	0
		(V, III)	5	1	0	6
		(V, IV)	0	0	0	0
		(V, V)	0	0	0	0

ICG DBF, indocyanine green dermal backflow
